# Site-Specific Bioconjugation of a Murine Dihydrofolate Reductase Enzyme by Copper(I)-Catalyzed Azide-Alkyne Cycloaddition with Retained Activity

**DOI:** 10.1371/journal.pone.0098403

**Published:** 2014-06-02

**Authors:** Sung In Lim, Yukina Mizuta, Akinori Takasu, Yong Hwan Kim, Inchan Kwon

**Affiliations:** 1 Department of Chemical Engineering, University of Virginia, Charlottesville, Virginia, United States of America; 2 Department of Frontier Materials, Nagoya Institute of Technology, Nagoya, Aichi, Japan; 3 Department of Chemical Engineering, Kwangwoon University, Seoul, Republic of Korea; 4 School of Materials Science and Engineering, Gwangju Institute of Science and Technology (GIST), Gwangju, Republic of Korea; University of Melbourne, Australia

## Abstract

Cu(I)-catalyzed azide-alkyne cycloaddition (CuAAC) is an efficient reaction linking an azido and an alkynyl group in the presence of copper catalyst. Incorporation of a non-natural amino acid (NAA) containing either an azido or an alkynyl group into a protein allows site-specific bioconjugation in mild conditions via CuAAC. Despite its great potential, bioconjugation of an enzyme has been hampered by several issues including low yield, poor solubility of a ligand, and protein structural/functional perturbation by CuAAC components. In the present study, we incorporated an alkyne-bearing NAA into an enzyme, murine dihydrofolate reductase (mDHFR), in high cell density cultivation of *Escherichia coli*, and performed CuAAC conjugation with fluorescent azide dyes to evaluate enzyme compatibility of various CuAAC conditions comprising combination of commercially available Cu(I)-chelating ligands and reductants. The condensed culture improves the protein yield 19-fold based on the same amount of non-natural amino acid, and the enzyme incubation under the optimized reaction condition did not lead to any activity loss but allowed a fast and high-yield bioconjugation. Using the established conditions, a biotin-azide spacer was efficiently conjugated to mDHFR with retained activity leading to the site-specific immobilization of the biotin-conjugated mDHFR on a streptavidin-coated plate. These results demonstrate that the combination of reactive non-natural amino acid incorporation and the optimized CuAAC can be used to bioconjugate enzymes with retained enzymatic activity.

## Introduction

Enzymes play important roles in biocatalysis to produce value-added compounds, as well as in the diagnostics and therapeutics to improve human health. In order to further expand the utility of enzymes, bioconjugation has been actively explored. The conjugation of polyethylene glycol (pegylation) to therapeutic enzymes leads to prolonged circulation time in vivo [1]. The conjugation of fluorescence dye facilitates the studies on protein trafficking inside cells/tissues or protein structural changes [2]–[4]. The covalent attachment of enzymes on solid surface (enzyme immobilization) leads to an enhanced thermostability [5]. For these applications, amino acids with reactive residues such as lysine and cysteine have been common targets for bioconjugation [6], [7]. However, the bioconjugation to multiple lysine residues of an enzyme often leads to heterogeneous mixtures of isomers, compromising the catalytic properties probably due to modification of an active site. In order to overcome this issue, the site-specific bioconjugation of an enzyme has been investigated. Although cysteine can be used to achieve site-specific bioconjugation in some cases, its application is limited in cases where there is an additional free cysteine residue or a disulfide bond(s) is critical for protein folding. In order to achieve absolute site-specificity, we and several other groups employed the bioconjugation strategy utilizing site-specific incorporation of a reactive non-natural amino acid (NAA) [8]–[11]. To attain an efficient site-specific incorporation, an expression host is equipped with an orthogonal pair of suppressor tRNA and aminoacyl-tRNA synthetase that have been modified to be specific for a NAA of interest but not to cross-talk with 20 natural amino acids as well as endogenous sets of tRNA and tRNA synthetase [12], [13]. The orthogonal pair incorporates a NAA in response to an expanded genetic code, usually stop codons or four-base codons [14], [15]. In particular, site-specific incorporation of a NAA is of interest for protein bioconjugation, because it allows a flexible selection of an incorporation site and subsequent chemo-selective conjugation, affording a conjugate with high homogeneity and minimal loss of native protein function.

In order to achieve the site-specific bioconjugation using a NAA, bioorthogonal protein chemistry is also required. We chose Cu(I)-catalyzed azide-alkyne cycloaddition (CuAAC) in this study, since it is a high-yielding and reliable reaction forming a stable triazole linkage between biologically inert azide and terminal alkyne groups [16], and readily compatible with aqueous and mild conditions beneficial for protein bioconjugation [17]–[19]. CuAAC has been embraced for numerous biomolecular conjugation applications since its discovery in 2002 [20]–[23]. To employ CuAAC, a NAA functionalized with an azide or an alkyne group is incorporated into a target protein in a residue- or a site-specific manner [11], [24], [25].

Efficient protein bioconjugation has been the primary goal for optimizing CuAAC conditions generating varying compositions of reaction components [26]–[31]. Although the retained stability of a viral capsid protein and the fluorescence of superfolder fluorescent protein after bioconjugation were investigated [11], [28], [32], only limited investigations have been performed where the retention of catalytic activity has been explicitly addressed. It was reported that a lipase conjugated to nanoparticles via CuAAC exhibited catalytic activity [33]. However, the extent of activity retention upon the conjugation was not presented. Since CuAAC reaction components or conditions may damage catalytic properties of enzymes, the CuAAC conditions needed to achieve a high bioconjugation yield may not be ideal for the bioconjugation of enzymes, while retaining their activity. For instance, *Candida antarctica* lipase B exhibited significant loss of activity when incubated overnight with CuAAC reagents including CuSO_4_, ascorbate, and bathophenanthroline ligand [34]. More recently, when *Escherichia coli* dihydrofolate reductase with a site-specifically incorporated tyrosine analog was subjected to direct protein-protein conjugation through CuAAC, and the authors reported complete loss of activity resulting from detrimental effects of the Cu(I) complex [35]. Considering the great potential of CuAAC in bioconjugation of enzymes and therapeutic proteins, it is timely and important to establish whether CuAAC conditions can be optimized to efficiently bioconjugate enzymes with retained enzymatic activity.

As a model system to study site-specific bioconjugation of enzymes via CuAAC, we chose murine dihydrofolate reductase enzyme (mDHFR), an enzyme that converts dihydrofolate (DHF) to tetrahydrofolate (THF). Here we first evaluated effects of CuAAC reaction components on retention of the catalytic activity as well as reaction efficiency in order to identify optimal CuAAC reaction conditions for enzyme bioconjugation. Furthermore, varying bioconjugation yields at different conjugation sites were also investigated. Since not all residues of an enzyme are readily accessible for bioconjugation, a choice of conjugation site is important to achieve high bioconjugation yield. Based on the solvent accessibility of amino acid residues in the mDHFR calculated by the ASA-View program [36], two residues (one with a high solvent accessibility and another with a low solvent accessibility) were selected. Then, the conjugation yield at the two sites was compared. Finally, with the optimized CuAAC conditions and conjugation site, we investigated whether an enzyme can be directly immobilized on the surface with preserved catalytic activity. Directed immobilization of a protein is one important topic in the fields of biosensing and biocatalysis, because it may increase the biosensor's sensitivity and biocatalyst's stability [37], [38]. In order to mediate enzyme immobilization, a biotin/streptavidin pair with strong affinity and high selectivity was utilized. A biotin derivative containing an azide functional group was site-specifically conjugated to the mDHFR and then subjected to binding to a streptavidin-coated plate.

## Results and Discussion

### Incorporation of *p*-Ethynylphenylalanine into the mDHFR

**Figure 1:**
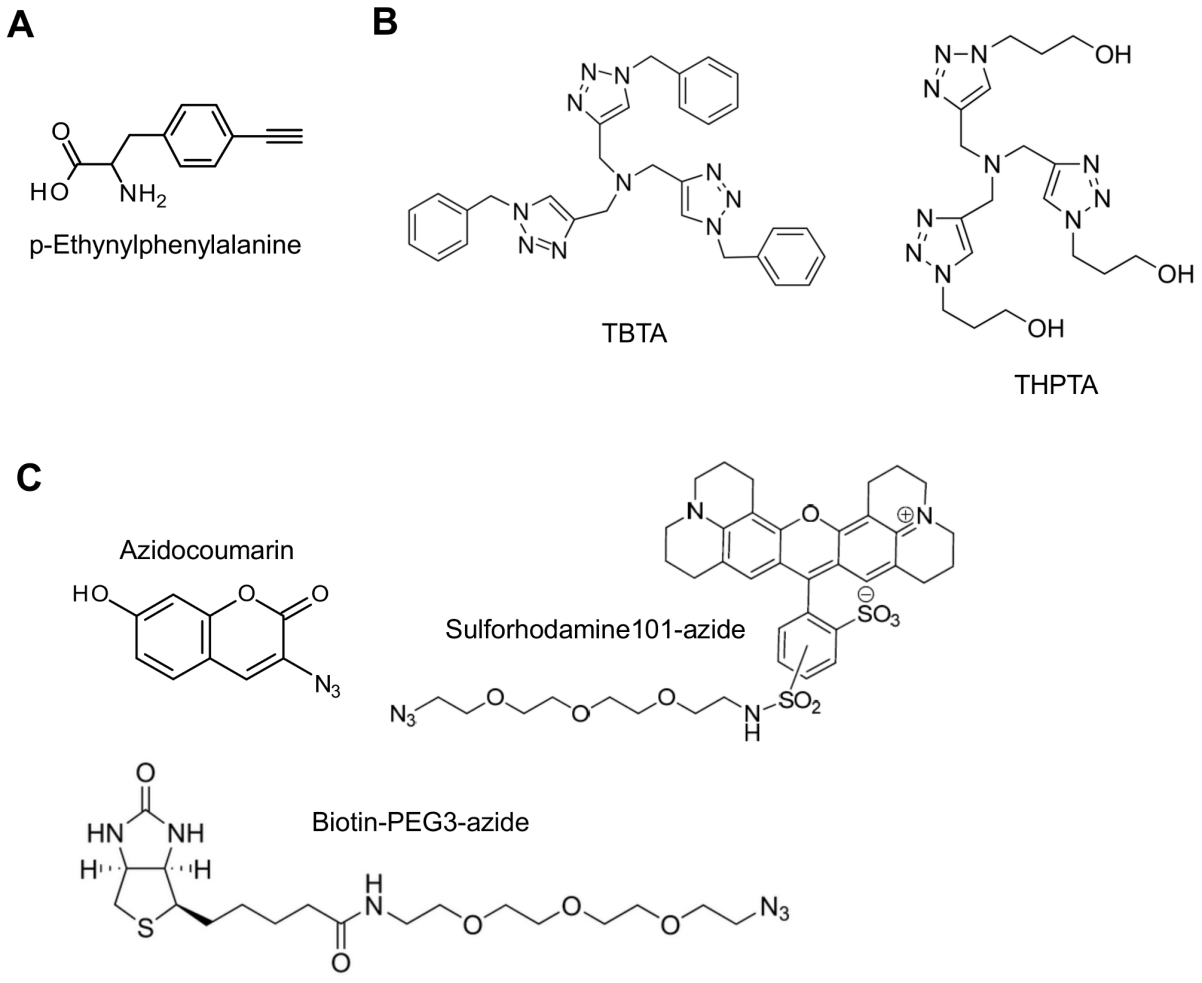
Chemical structures. (A) *p*-ethynylphenylalanine, (B) Cu(I)-chelating ligands, and (C) azide-functionalized reagents.

**Figure 2:**
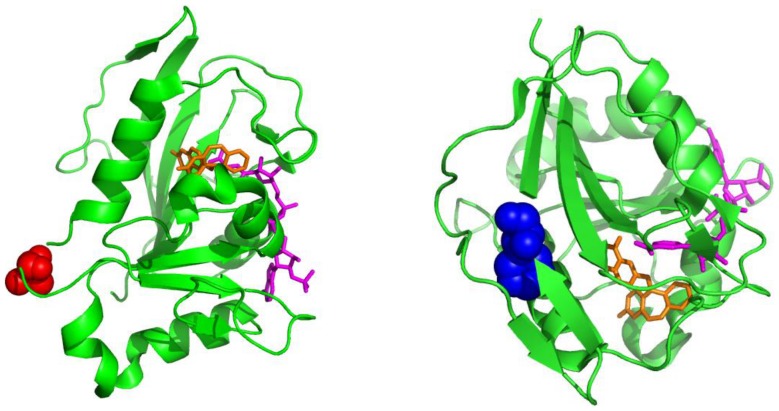
Locations of selected residues for pEthF incorporation in the three-dimensional structure of the mDHFR-WT. Valine at the 43rd position and phenylalanine at the 179th position are highlighted in red (left) and blue (right), respectively. The cofactor NADPH (magenta) and the inhibitor (orange) are shown in stick representation (PDB ID: 3D80).

To minimize structural perturbation upon incorporation of hydrophobic phenylalanine analog, *p*-ethynylphenylalanine (pEthF) (Figure 1A), the valine at the 43rd position was chosen as a target because it is away from the active site (Figure 2), and exhibits hydrophobic index similar to that of phenylalanine [39] and substantial solvent accessibility (Figure S1A). Residues were numbered based on the amino acid sequence of the mDHFR in the Protein Data Bank (PDB ID: 3D80) [40]. We introduced an amber codon at the 43^rd^ position of the mDHFR to replace valine with pEthF (mDHFR-43pEthF). Protein expression in a site-specific incorporation system generally suffers from low protein yield because an exogenous suppressor tRNA should compete with endogenous release factor 1 for the recognition of an amber stop codon [41], necessitating a large volume of culture to secure an appreciable amount of a target protein. In addition, a high NAA concentration, typically in mM range, is required to maximize the fraction of a suppressor tRNA charged with a NAA [42], [43], which is expensive or commercially not available. To increase expression yield of the mDHFR containing a site-specifically incorporated pEthF with its minimal consumption, cells were harvested by centrifugation before IPTG induction, and then resuspended with M9 expression medium containing 3 mM pEthF, the volume of which was 20-fold less than the original volume. Previously, condensed *E. coli* cultures in the NAA incorporation system employing an evolved pyrrolysyl-tRNA synthetase/tRNA_CUA_ pair from *Methanosarcina* species significantly enhanced the expression yield of proteins containing a NAA [44], [45]. The methodology has been found to be effective with the yeast phenylalanyl-tRNA synthetase/suppressor tRNA machinery we used here. When the culture volume was condensed by a factor of 20 but the pEthF concentration was not changed, the amount of the mDHFR-43pEthF obtained per milligram of pEthF (65.2 µg) was approximately 19-fold higher than that in the uncondensed culture (3.5 µg). These results successfully demonstrate that the production of the mDHFR-pEthF in a condensed volume of cell culture greatly minimizes the waste of a valuable NAA.

**Figure 3:**
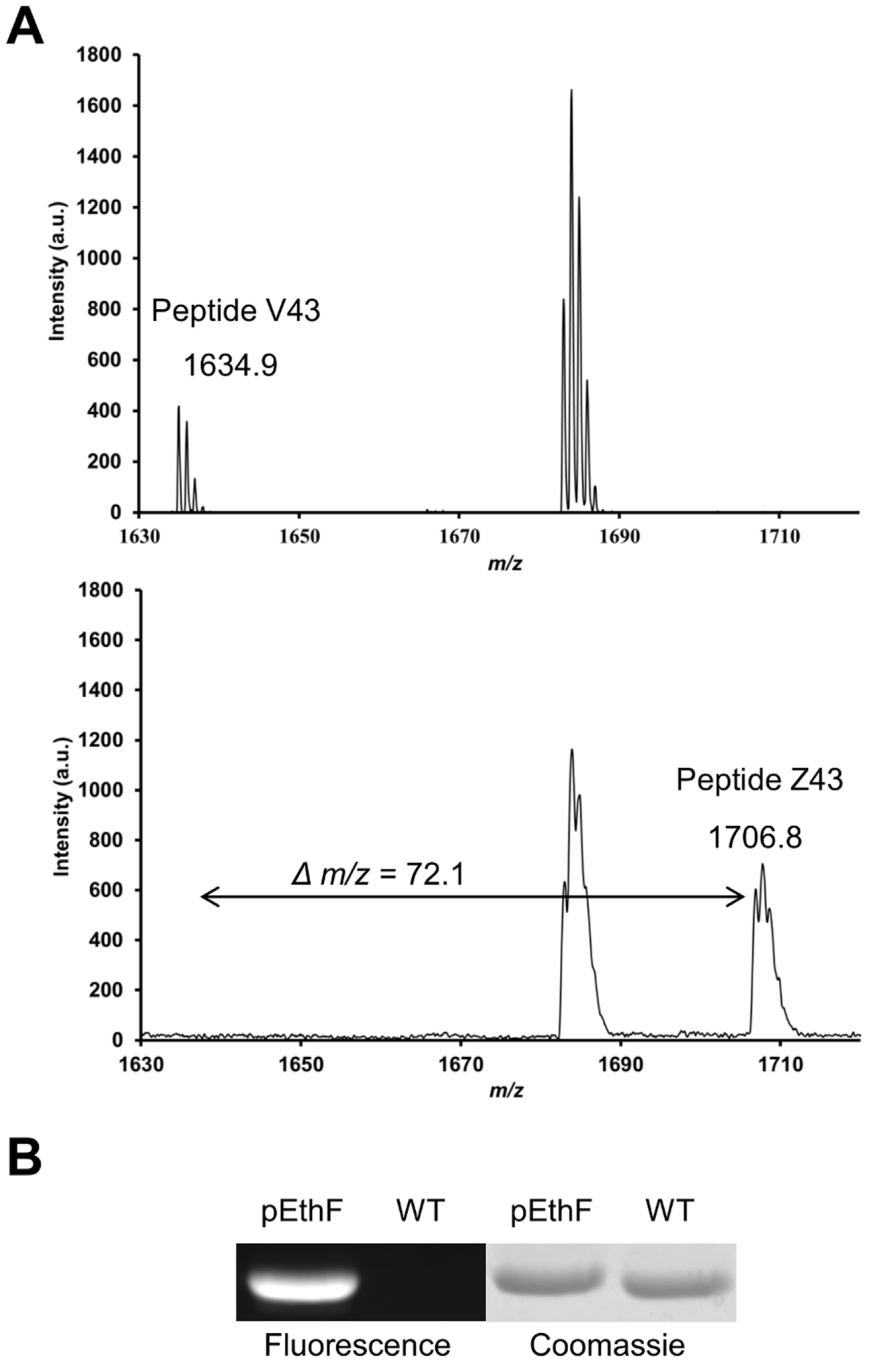
Incorporation of pEthF into the 43rd position of the mDHFR and its orthogonal reactivity. (A) MALDI-TOF spectra of Lys-C-digested fragments derived from the mDHFR-WT (top) and the mDHFR-pEthF (bottom). (B) SDS-PAGE of the mDHFR-pEthF (pEthF) and the mDHFR-WT (WT) reacted with sulforhodamine-azide. The reaction was performed at 25°C by mixing the protein (30 µM) with the dye (60 µM), CuSO_4_ (1 mM), TBTA (1 mM), and ascorbate (2 mM) in 20 mM phosphate (pH 8.0) plus 30% (v/v) DMSO. The gel was illuminated by the excitation light at 550 nm (fluorescence panel), and then stained with Coomassie brilliant blue (Coomassie panel).

To verify the substitution of the 43rd valine by pEthF, endoproteinase Lys-C digests of the mDHFR-43pEthF and the wild-type mDHFR (mDHFR-WT) were analyzed by MALDI-TOF. Peptide V43 (residue 33–46; YFQRMTTTSS**V**EGK) derived from the mDHFR-WT, was detected with a monoisotopic mass of 1634.9 Da, in accord with its theoretical mass (Figure 3A, top). For the mDHFR-43pEthF, Peptide Z43 (residue 33–46; YFQRMTTTSS**Am**EGK where Am indicates an amber codon) was observed at 1706.8 Da, while no signal was found at 1634.9 Da (Figure 3A, bottom), strongly supporting the incorporation of pEthF in response to the amber codon.

To validate orthogonal reactivity of the alkynyl group at the para-position of pEthF, the mDHFR-43pEthF, along with the mDHFR-WT as a control, was incubated with a fluorescent probe, sulforhodamine-azide (Figure 1C), in the presence of CuSO_4_, ascorbate, and TBTA. In-gel fluorescence analysis showed that only the mDHFR-43pEthF was reactive toward the azide group via CuAAC (Figure 3B).

### Effect of CuAAC in TBTA/DMSO System on Enzymatic Activity

The catalytic function of an enzyme is sensitive to the chemical environment, and often severely hampered by suboptimal reaction conditions. There are several reasons for such a loss of catalytic activity. First, the addition of DMSO or SDS to solvate hydrophobic surfaces and chelating ligands may lead to irreversible deformation of the three-dimensional structure [26], [28], [45]. Second, harmful byproducts formed from a reductant can adversely modify proteins [28], [46]. Finally, lack of CuAAC kinetic studies when a protein serves as a reaction target obscures an optimal reaction time that meets both maximum yield and minimal exposure to the abovementioned potential risk factors. Therefore, to ensure enzyme-friendly applications of CuAAC, proper choices of the chelating ligand, the reductant, and duration of reaction should be addressed.

**Figure 4:**
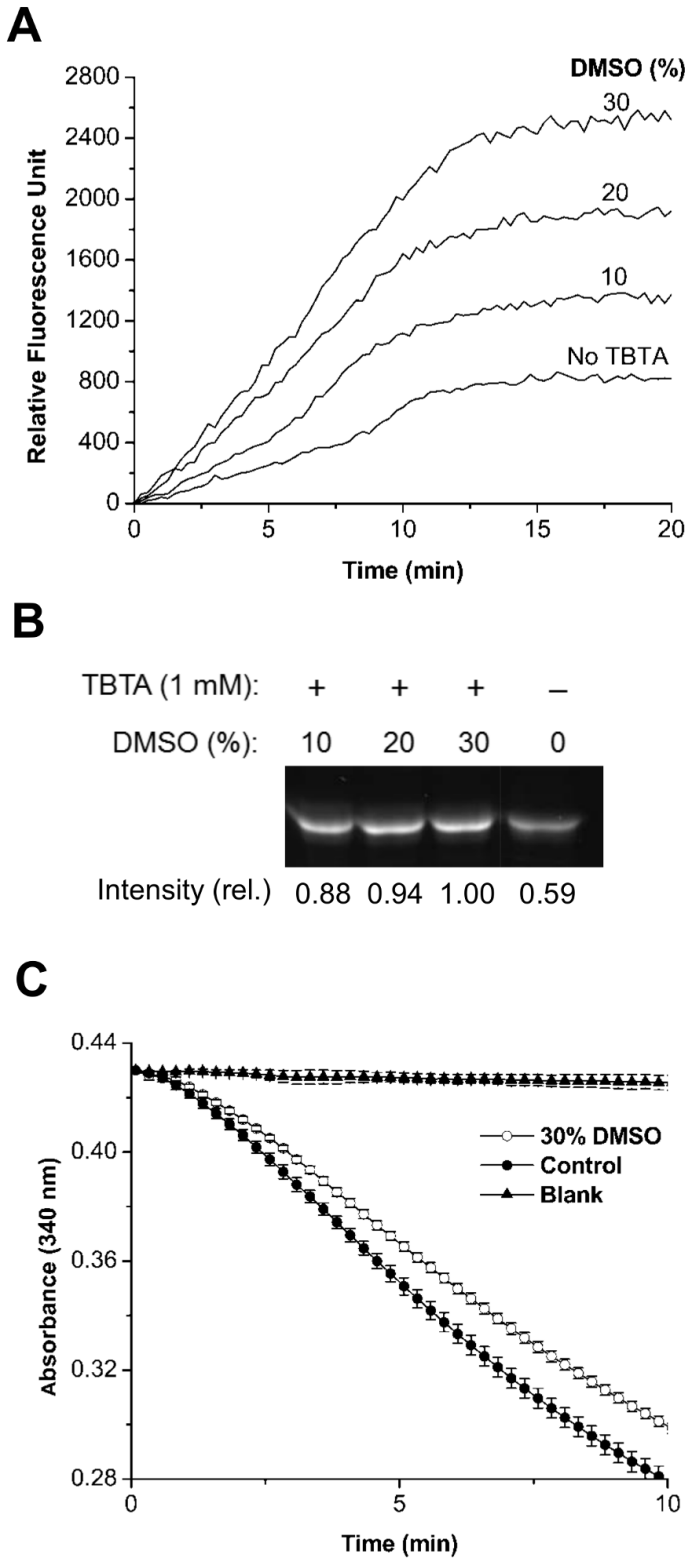
Performance of TBTA-assisted CuAAC reactions under various DMSO concentrations and their effects on the enzymatic activity. (A) Kinetic traces detected at various DMSO volume concentrations in the presence of TBTA or in a DMSO-free buffer without TBTA. Reactions were initiated at 25°C by adding ascorbate (2 mM) to a phosphate-buffered (pH 8.0) mixture containing 30 µM of the mDHFR-43pEthF, 60 µM of azidocoumarin, 1 mM of CuSO_4_, 1 mM TBTA, and appropriate DMSO contents. Fluorescence evolution was recorded at λ_ex_ = 400 nm and λ_em_ = 470 nm. (B) In-gel fluorescence of an equal amount of reaction products. Relative intensities were quantified by densitometry. (C) Effect of DMSO incubation on activity of the mDHFR-43pEthF. After incubation of 30 µM protein in 10 µl of DMSO-free (closed circle) or 30% (v/v) DMSO-containing phosphate buffer (pH 8.0) (open circle) for 15 min at 25°C, the mixture was diluted 36-fold with the assay buffer for activity assay as described in Materials and Methods. Relative activity was calculated based on changes in absorbance for 10 min after initiation of the enzymatic reaction. Error bars represent standard errors (n = 3).

TBTA (Figure 1B) stabilizes Cu(I) ions which are susceptible to disproportionation, and has been commonly used as an accelerating ligand for CuAAC. However, due to its low water-solubility, it tends to become precipitated in aqueous media in which most protein conjugations are conducted. To increase TBTA solubility, we incrementally added a polar organic solvent, DMSO, to the CuAAC reaction mixture while holding all others fixed, and explored its effect on reaction yield. A milky turbidity observed in the CuAAC reaction mixture dropped in proportion to DMSO concentration. In order to evaluate reaction efficiency, we employed fluorogenic assay using azidocoumarin (Figure 1C) which, initially non-fluorescent, becomes highly fluorescent upon its conjugation to terminal alkyne through CuAAC, thereby enabling spectroscopic tracking of the reaction progress [47]. Higher DMSO concentration allowed higher conjugation efficiency, indicating that solubility of TBTA is important to a high-yielding CuAAC reaction (Figure 4A). DMSO-dependent labeling efficiency was also evident by in-gel fluorescence assay (Figure 4B). As quantified by fluorescence intensity, the CuAAC reaction in 30% (v/v) DMSO exhibited 13.6% stronger fluorescence than that in 10% (v/v) DMSO.

It was previously reported that DMSO exerts inhibitory effects on catalytic enzymes [48]–[50]. In particular, *E. coli* dihydrofolate reductase lost 17% of its native enzymatic activity after incubation in the CuAAC reaction buffer containing 20% (v/v) DMSO [51]. To investigate the effect of DMSO on enzymatic activity of the mDHFR-43pEthF, we measured NADPH-dependent reduction of DHF catalyzed by the mDHFR-43pEthF after 15 min incubation with 30% (v/v) DMSO and 36-fold dilution with the assay buffer (Figure 4C). The mDHFR-43pEthF exhibited 86.7% of its original activity after incubation, indicating that 30% (v/v) DMSO essential for maximum yield irreversibly impairs the enzymatic activity and, therefore, the TBTA/DMSO system for CuAAC is not compatible with the mDHFR.

### CuAAC with the Water-soluble Chelating Ligand, THPTA

**Figure 5:**
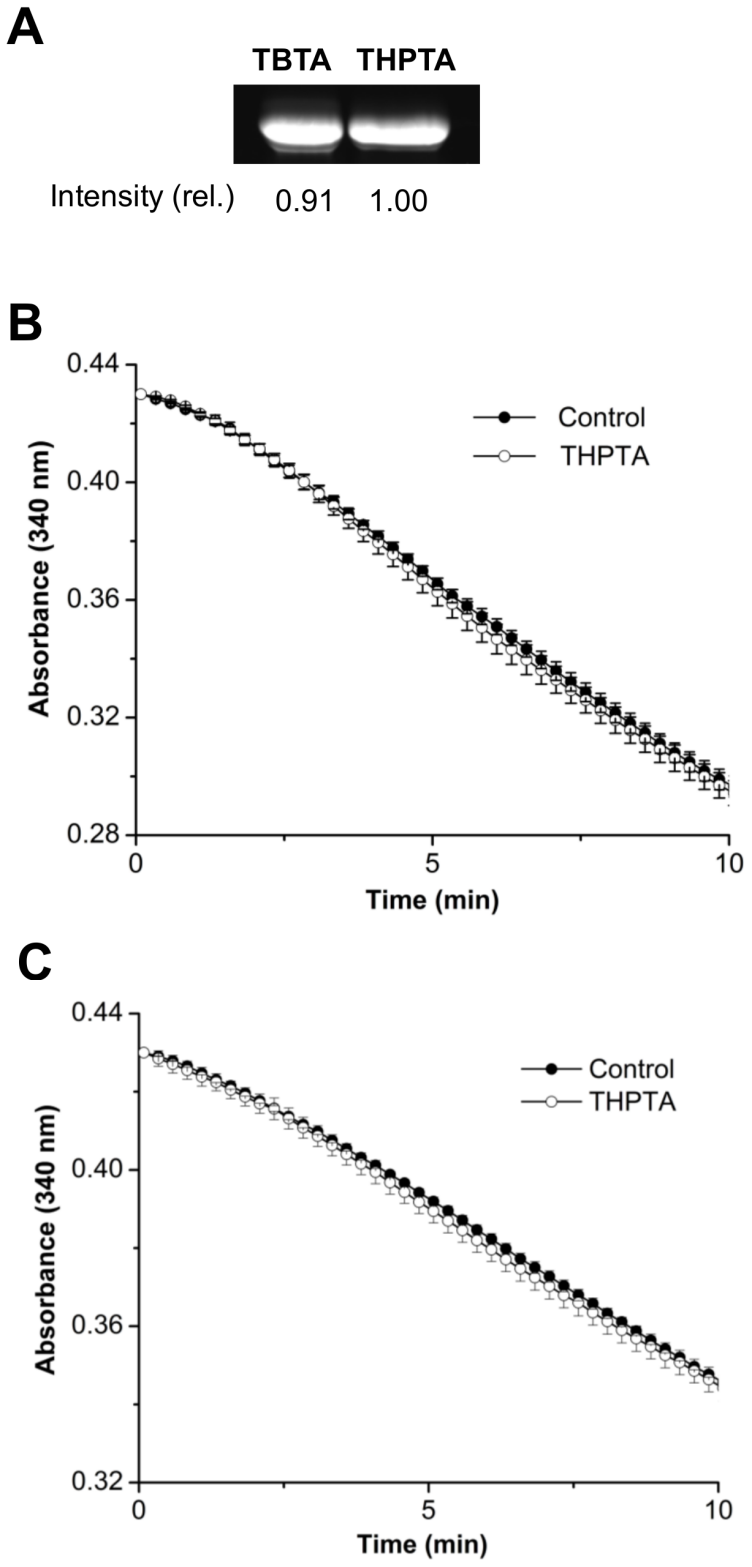
THPTA-assisted CuAAC reaction and its effect on the enzymatic activity. (A) In-gel fluorescence of reaction products from TBTA- and THPTA-assisted dye labeling via CuAAC. The mDHFR-pEthF (30 µM) was reacted at 25°C for 15 min with sulforhodamine-azide (60 µM) in the presence of 1 mM CuSO_4_, 1 mM TBTA, 2 mM ascorbate in a phosphate buffer (pH 8.0) containing 30% (v/v) DMSO or in the presence of 1 mM CuSO_4_, 1 mM THPTA, 2 mM ascorbate in a DMSO-free phosphate buffer (pH 8.0). (B) Effect of THPTA incubation on activity of the mDHFR-43pEthF. After incubation of 30 µM protein with (open circle) or without (close circle) 1 mM THPTA in 10 µl of DMSO-free phosphate buffer (pH 8.0) for 15 min at 25°C, the mixture was diluted 36-fold with the assay buffer for activity assay. Error bars represent standard errors (n = 3). (C) Effect of THPTA incubation on activity of the mDHFR-179pEthF (n = 3).

To circumvent the use of DMSO, which is essential for TBTA-mediated CuAAC but adversely affects the enzymatic activity, we employed a water-soluble ligand, THPTA (Figure 1B) [52], and tested its performance as a chelating ligand. At the same concentration of 1 mM, the CuAAC labeling using THPTA in an aqueous buffer was as efficient as the labeling using TBTA in 30% DMSO (Figure 5A). In contrast to the TBTA/DMSO system, the catalytic activity of the mDHFR-43pEthF was preserved after incubation with THPTA in DMSO-free reaction buffer (Figure 5B). The same condition could be applied to the other mDHFR mutant bearing a site-specifically incorporated pEthF at the 179th position (mDHFR-179pEthF) without harming its native activity (Figure 5C). These results indicate that THPTA is a relevant substitute for TBTA as a chelating ligand for enzyme bioconjugation via CuAAC.

### Optimization of CuAAC with Various Reductants

**Figure 6:**
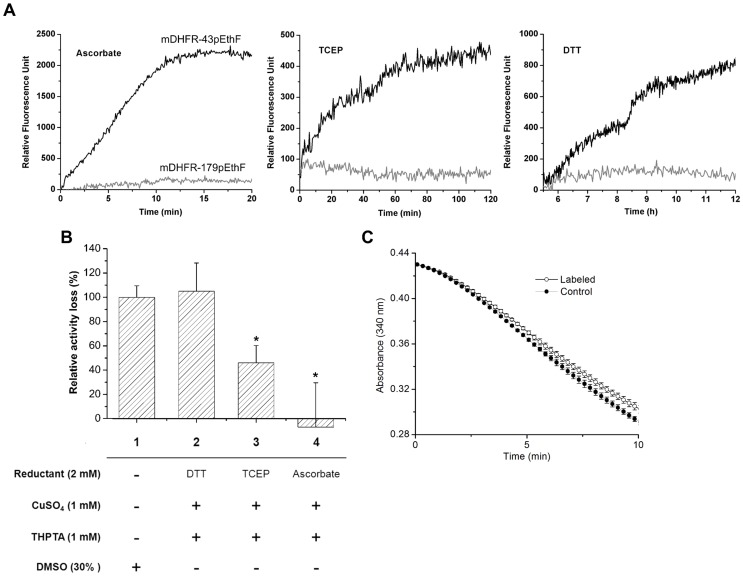
Effect of various reductants on CuAAC reaction rates and activities of the mDHFR-43pEthF and the mDHFR-179pEthF. (A) Time course of CuAAC reactions initiated by ascorbate, TCEP, and DTT. Reactions were performed at 25°C by adding 2 mM reductant to a phosphate-buffered (pH 8.0) mixture containing 30 µM of the mDHFR-43pEthF (black) or the mDHFR-179pEthF (gray), 60 µM of azidocoumarin, 1 mM of CuSO_4_, 1 mM THPTA. Fluorescence evolution was recorded at λ_ex_ = 400 nm and λ_em_ = 470 nm. (B) Relative loss of enzymatic activity after incubation with various CuAAC systems in the absence of azidocoumarin (**1**–**4**). Incubation times were 15 min for system **1** and **4**, 12 h for system **2**, and 2 h for system **3**. Activity losses were normalized to that in system **1**. Error bars represent standard errors (n = 3). Two-sided Student's t-tests were applied to the data (*P<0.05). (C) Effect of ascorbate-driven dye labeling of the mDHFR-43pEthF on the enzymatic activity. The labeling was performed at 25°C for 15 min in the presence of 30 µM of the mDHFR-43pEthF, 60 µM of azidocoumarin, 1 mM of CuSO_4_, 1 mM THPTA, and 2 mM ascorbate. Error bars represent standard errors (n = 3).

We explored relative compatibility of various reductants in terms of their effects on the reaction rate and enzymatic activity of the mDHFR-43pEthF. We chose three reductants: tris (2-carboxyethyl)phosphine (TCEP), a highly water-soluble and relatively stable reductant [46]; ascorbate, a commonly used reductant in CuAAC but known to produce unwanted byproducts at certain conditions [53], [54]; and dithiothreitol (DTT), a strong reductant whose use in CuAAC was recently reported [55]. First, we labeled the mDHFR-43pEthF with a fluorogenic probe, azidocoumarin, using each reductant, and measured the time-course evolution of fluorescence on a microwell plate (Figure 6A). Ascorbate was shown to be the most powerful reductant, completing the reaction within 15 min with the highest fluorescence at the plateau. The yield was substantially lower when TCEP or DTT was used. Moreover, the time to completion was prolonged to more than 2 h and 12 h, respectively. Even though ascorbate seemed most effective in reaction kinetics, it does not necessarily mean that it has the best compatibility. To assess an adverse effect of CuAAC systems on enzymatic activity, the mDHFR-43pEthF was incubated with each system, and then subjected to the activity assay (Figure 6B). Incubation times were set at 15 min, 2 h, and 12 h, i.e. time for reaction completion, for ascorbate-, TCEP- and DTT-reducing system, respectively. Considering large differences in the conjugation yield observed among the reaction conditions, the mDHFR was incubated in the absence of any dye. The relative reduction in activity was greatest in DTT-reducing system, followed by TCEP- and ascorbate-reducing system. Compared to the activity loss observed after incubation with 30% DMSO, TCEP- and ascorbate-reducing system showed significantly (P<0.05) lower reduction in activity. Notably, the mDHFR-43pEthF treated with ascorbate did not cause any significant reduction in activity compared to that of the untreated control. These results suggest that short incubation with the reductant is critical to minimize adverse effects on enzymatic activity during CuAAC reaction, and ascorbate is the best reductant for CuAAC with an enzyme because it rapidly completes the reaction with high yield. Using the optimized CuAAC condition, an azidocoumarin was conjugated to the mDHFR-43pEthF and then the activity of the dye-conjugated mDHFR-43pEthF was compared to that of the untreated mDHFR-43pEthF (Figure 6C). As expected, the dye-conjugation led to only a minor reduction in activity, which is likely due to the slight structural perturbation resulting from the hydrophobic nature of the dye conjugated.

Previously, complete loss of enzymatic activity was observed upon bioconjugation of the *E. coli* DHFR variant in which the CuAAC reaction was initiated by adding a reactive Cu(I) complex, and incubated overnight [35]. Our optimization study therefore suggests a remedy to implement enzyme-compatible bioconjugation through in situ reduction of Cu(II) by an appropriate choice of a reductant allowing minimum contact time between the enzyme and reactive species while achieving maximum conjugation yield. Although the CuAAC condition described here works well for the mDHFR, it should, however, be applied to other enzymes with a caution. Considering the physicochemical diversity of enzymes and varying sensitivities to reaction components (such as strong inhibition of formate dehydrogenase by copper ions), the CuAAC conditions may need to be slightly adjusted for each enzyme. In case of enzymes greatly inhibited by copper ions, copper-free azide-alkyne cycloaddition can be employed. The construction of multifunctional enzyme complex via copper-free azide-alkyne cycloaddition was performed leading to varying retained activities between 17 to 91% [56].

### Comparison of Conjugation Yields at Different Conjugation Sites

To compare the conjugation yield depending on the solvent accessibility of a conjugation site, the phenylalanine at position 179 (F179) was also targeted for pEthF incorporation generating the mDHFR-179pEthF (Figure 2 and Figure S1B). Contrary to the solvent accessible valine at position 43 (accessibility score: 0.44), a phenyl ring of F179 is buried inside the protein and has a very low solvent accessibility (accessibility score: 0.10). Under the same condition used for the mDHFR-43pEthF, fluorescence signals of the mDHFR-179pEthF were substantially lower than those of the mDHFR-43pEthF (Figure 6A). These results strongly indicate that the solvent accessibility of a conjugation site correlates to the conjugation yield. When the crystal structure of a target protein is available, the solvent accessibility prediction tools including ASA-View program can be used to identify a suitable conjugation site leading to a high conjugation yield. Furthermore, in the absence of the protein crystal structure, monitoring fluorogenic dye conjugation at each site is expected to be used to estimate the solvent accessibility. It should be also noted that the mDHFR-179pEthF had only 30% of original activity of the mDHFR (data not shown). It is known that F179 stabilizes the tertiary structure of the mDHFR by forming a parallel ring stacking interaction with Y33 [57]. Its substitution by even a structurally similar phenylalanine analog appears to adversely impact the aromatic ring stacking, thereby resulting in distorted conformation for ligand binding.

### Immobilization of the mDHFR-43pEthF by Site-specific Biotinylation

**Figure 7:**
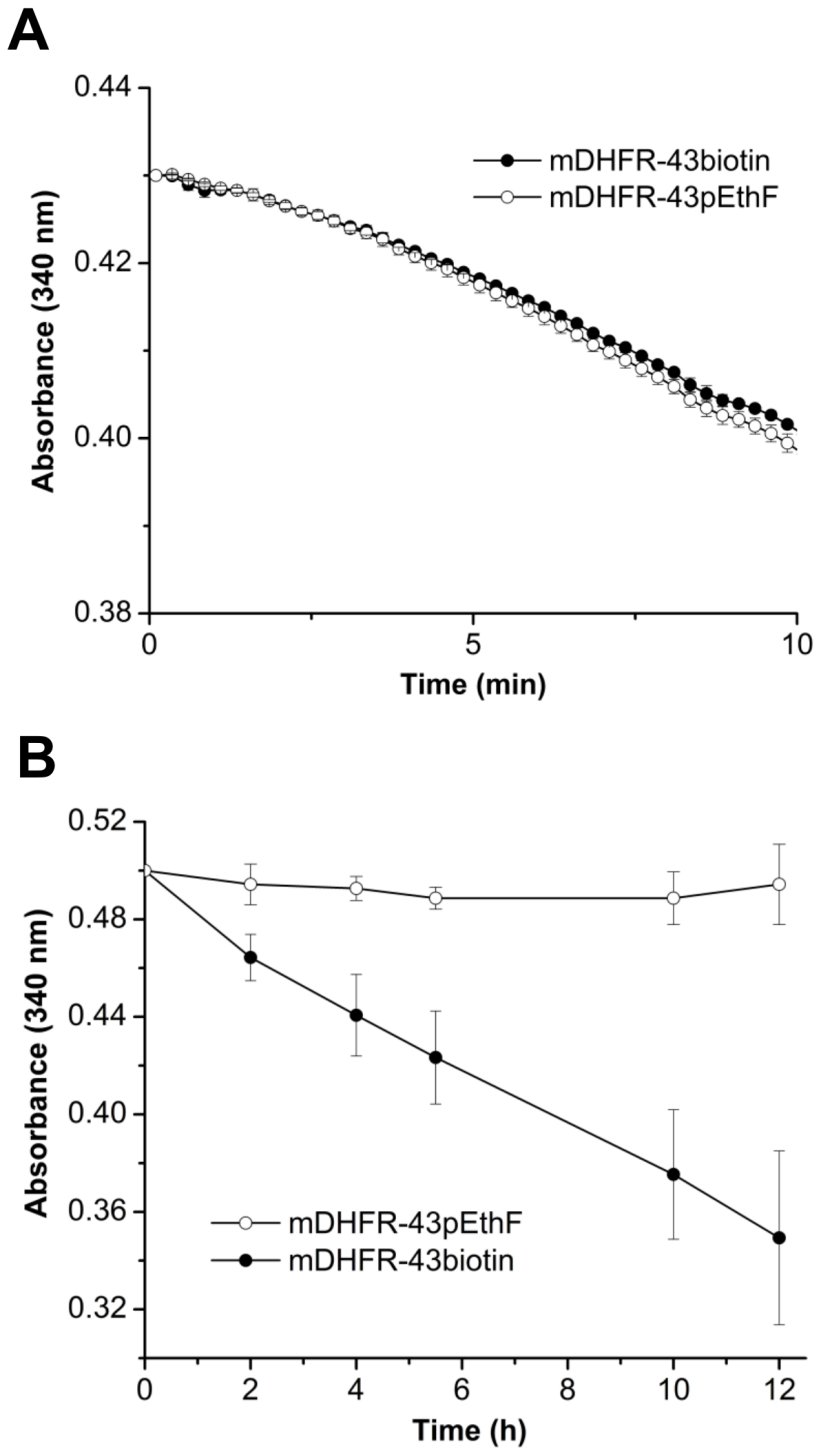
Enzymatic activity of the mDHFR after site-specific biotinylation and immobilization. (A) Activity of the mDHFR-43biotin versus the mDHFR-43pEthF. Error bars represent standard errors (n = 3). (B) Activity of the immobilized mDHFR-43biotin. Streptavidin-coated wells were incubated with 50 µL of the mDHFR-43biotin and the mDHFR-43pEthF at 1 mg/mL, separately, for 30 min at RT. After washing, the enzymatic reaction was initiated at 25°C by adding 200 µL of assay buffer, and monitored by spectrometry. Error bars represent standard errors (n = 3).

As a practical application of site-specific bioconjugation of an enzyme, we investigated if the mDHFR can be immobilized onto a streptavidin-coated plate through CuAAC-mediated biotinylation without loss of activity. Biotin-PEG3-azide, a hybrid reagent having a flexible and hydrophilic PEG spacer and a chemoselective azide group, was conjugated to the mDHFR-43pEthF in the ascorbate-reducing system to yield the biotinylated mDHFR (mDHFR-43biotin). Little change in enzymatic activity was observed for the mDHFR-43biotin in comparison to the mDHFR-43pEthF, suggesting that biotinylation as well as the reaction system did not seriously interfere with the activity (Figure 7A). Biotinylation was found to be high yielding as revealed by the dye-conjugation analysis (Figure S2). The inertness of the mDHFR-43biotin towards the CuAAC-driven dye labeling indicated that most of the accessible pEthFs had been occupied by the biotin. To implement enzyme immobilization through biotin-streptavidin interaction, the streptavidin-coated plate was incubated with the mDHFR-43biotin, in parallel with the mDHFR-43pEthF as a control (Figure 7B). Positive enzymatic activity was detected over course of time, but not in the control. To our knowledge, this is the first example of enzyme immobilization through site-specific NAA incorporation and the CuAAC with retained catalytic activity, and opens the possibility to fabricate highly sensitive biosensors and biocatalysts attributed by controlled orientation and homogeneous chemistry.

## Conclusions

Site-specific bioconjugation of an enzyme was achieved by introducing a NAA with an alkyne functional group into a specific site followed by conjugation via CuAAC. The effects of CuAAC conditions on catalytic activity were evaluated using the mDHFR as a model enzyme. Under the optimized CuAAC condition utilizing THPTA and ascorbate as a ligand and a reductant, respectively, the fluorescence dye and biotin were efficiently conjugated to the mDHFR in a site-specific manner with retention of substantial catalytic activity. It was also found that the solvent accessibility of a conjugation site correlates to a conjugation yield indicating that the choice of conjugation site is critical to ensure efficient bioconjugation. Furthermore, the site-specific biotinylation was successfully used to immobilize the mDHFR on a streptavidin-coated plate through a highly specific and tight biotin-streptavidin binding, which would enable a spatially controlled and maximally functional enzyme immobilization.

## Materials and Methods

### Materials

*p*-Ethynylphenylalanine (pEthF) was synthesized as described previously [58]. Ni-NTA agarose and pQE16 plasmid were obtained from Qiagen (Valencia, CA). Endoproteinase Lys-C was obtained from Promega Corporation (Madison, WI). Amicon ultra centrifugal filters with a molecular weight cutoff of 10 kDa and ZipTip with C_18_ media were purchased from Millipore Corporation (Billerica, MA). Sulforhodamine-azide and Biotin-PEG3-azide were purchased from Bioconjugate Technology Company (Scottsdale, AZ). Azidocoumarin was obtained from Glen Research (Sterling, VA). Streptavidin Coated High Sensitivity Plate was obtained from Thermo Scientific (Rockford, IL). All other chemicals were purchased from Sigma-Aldrich Corporation (St. Louis, MO).

### Plasmid Construction and Strains

Preparation of the plasmids pQE16-yPheRS^T415A^ and pREP4-ytRNA^Phe^_CUA_UG_ is described elsewhere [59]. pQE16_am43_-yPheRS^T415A^ or pQE16_am179_-yPheRS^T415A^ encodes the yeast phenylalanyl-tRNA synthetase variant and the murine dihydrofolate reductase (mDHFR) with an amber codon at the 43rd or 179th position and a C-terminal hexahistidine tag. An amber codon was introduced by PCR mutagenesis replacing the 43rd valine codon or the 179th phenylalanine codon. The mutagenic primer sequences were as follows: V43 Forw, 5′-CACAACCTCTTCATAGGAAGGTAAACAG-3′; V43 Rev, 5′- CTGTTTACCTTCCTATGAAGAGGTTGTG-3′; F179 Forw, 5′-CATCAAGTATAAGTAGGAAGTCTACGAG-3′; F179 Rev, 5′-CTCGTAGACTTCCTACTTATACTTGATG-3′. pREP4-ytRNA^Phe^_CUA_UG_ encodes the mutant yeast amber suppressor tRNA engineered to have minimal cross-reactivity with the *E. coli* aminoacyl-tRNA synthetases. A Phe/Trp/Lys triple auxotrophic *Escherichia coli* strain, AFWK, was prepared as described previously [59]. AFWK harboring both plasmids was used as an expression host for site-specific incorporation of pEthF into the amber codon.

### Expression and Purification of Proteins

The wild-type mDHFR (mDHFR-WT) was expressed from E. coli XL1-Blue harboring pQE16 by 1 mM IPTG induction in LB media containing 100 µg/mL ampicillin at 37 °C. To express the mDHFR mutant containing pEthF at the 43rd position (mDHFR-43pEthF), AFWK harboring pQE16_am43_-yPheRS^T415A^ and pREP4-ytRNA^Phe^_CUA_UG_ was used. Saturated overnight cultures grown at 37 °C in M9 minimal medium supplemented with 100 µg/mL ampicillin, 30 µg/mL kanamycin, 0.4% (w/v) glucose, 1 mM MgSO_4_, 0.1 mM CaCl_2_, 10 µg/mL thiamine, and 20 amino acids (25 µg/mL each) were diluted 20-fold in the same fresh medium, and grown at 37 °C until an OD_600_ of 0.9 was reached. After incubation on ice for 15 min, cells were sedimented by centrifugation at 4000 g for 12 min, and washed with cold 0.9% (w/v) NaCl by gentle resuspension. After repeating twice, cells were shifted to M9 medium supplemented with the same ingredients described above except for different amino acid compositions: 17 amino acids (35 µg/mL each), 150 µM Lys, 60 µM Phe, 20 µM Trp, and 3 mM pEthF. To maximize the incorporation efficiency in condensed culture, the total volume of M9 expression medium was 20-fold smaller than the original volume. Upon induction by 1 mM IPTG, cells were incubated with shaking at 30 °C for 15 h before harvest. Cells were pelleted by centrifugation, and the protein was purified by gravity-flow affinity chromatography using Ni-NTA agarose beads under native condition according to the supplier's instructions (Qiagen). Purified proteins were directly used or buffer-exchanged using PD-10 desalting columns to appropriate buffers. If necessary, the protein solutions were concentrated using centrifugal filters. The mDHFR mutant containing pEthF at the 179th position (mDHFR-179pEthF) was obtained as described above except that pQE16_am179_-yPheRS^T415A^ was used instead of pQE16_am43_-yPheRS^T415A^.

### Mass Characterization by MALDI-TOF Mass Spectrometry

To the mDHFR-WT or the mDHFR-pEthF in 20 mM potassium phosphate (pH 8.0) was added endoproteinase Lys-C to a final protease:mDHFR ratio of 1∶50 (w/w). Following incubation at 37 °C overnight, the reaction mixture was mixed with 0.5% (v/v) trifluoroacetic acid (TFA) to quench the reaction and then desalted on a ZipTip C_18_, and then analyzed by MALDI-TOF mass spectrometry (MS) to confirm site-specific incorporation of pEthF into the desired position of the mDHFR. The MS analysis was performed using 20 mg/mL of 2,5-dihydroxybenzoic acid and 2 mg/mL of *L*-(−)-fucose dissolved in 10% ethanol as a matrix by Microflex™ MALDI-TOF MS (Bruker Corporation, Billerica, MA).

### Kinetic Studies

Stock solutions were prepared as follows: 80 µM mDHFR-pEthF in 20 mM potassium phosphate (pH 8.0), 20 mM CuSO_4_ in DW, 20 mM tris[(1-benzyl-1H-1,2,3-triazol-4-yl)methyl]amine (TBTA) in dimethyl sulfoxide (DMSO), 20 mM tris(3-hydroxypropyltriazolylmethyl)amine (THPTA) in DW, 1 mM azidocoumarin in DMSO, and 40 mM reductant in DW. For CuAAC in the TBTA/DMSO system, the mDHFR-pEthF at a final concentration of 30 µM was mixed with an appropriate concentration of DMSO, 1 mM CuSO_4_, 1 mM TBTA, 60 µM of azidocoumarin, and 2 mM reductant in 70 µL reaction volume (listed in the order of addition). For CuAAC in the THPTA system, the mDHFR-pEthF at a final concentration of 30 µM was mixed with 1 mM CuSO_4_, 1 mM THPTA, 60 µM of azidocoumarin, and 2 mM reductant in 70 µL reaction volume. Evolution of fluorescence was monitored at λ_ex_ = 400 nm, λ_em_ = 470 nm in standard 96-well plates on the SynergyTM four multimode microplate reader (BioTek, Winooski, VT) at appropriate time intervals at 25°C. To quench the reaction, 10 mM ethylenediaminetetraacetic acid (EDTA) was added.

### Enzymatic Activity Assay

To measure enzymatic activity of the mDHFR-pEthF, nicotinamide adenine dinucleotide phosphate (NADPH)-dependent reduction of dihydrofolate (DHF) was monitored at A_340 nm_ by the SynergyTM four multimode microplate reader according to the protocol provided by the dihydrofolate reductase assay kit (Sigma) with slight modification as follows. The reaction was initiated by mixing 100 µL of the assay buffer (50 mM 2-(N-morpholino)ethanesulfonic acid (Mes), 25 mM tris(hydroxymethyl)aminomethane (Tris), 25 mM ethanolamine, and 100 mM sodium chloride, pH 7.5, containing 120 µM NADPH and 100 µM DHF) with 100 µL of the assay buffer containing an appropriate concentration of the mDHFR-pEthF. All measurements were made in triplicate at 25°C. The change in absorbance after 10 min was taken as a measure of catalytic activity.

### Immobilization and Activity Assay of the Biotinylated mDHFR

Biotinylation of the mDHFR-43pEthF was conducted for 20 min in the following condition: 50 µM mDHFR-43pEthF, 1 mM CuSO_4_, 1 mM THPTA, 150 µM of biotin-PEG3-azide, and 2 mM ascorbate in 20 mM potassium phosphate (pH 8.0)/0.1 M NaCl. After adding EDTA at a final concentration of 10 mM to quench the reaction, the reaction mixture was desalted by a PD-10 column, and concentrated by ultrafiltration to 1 mg/mL of the biotinylated mDHFR (mDHFR-43biotin). Fifty microliter of the mDHFR-43biotin was added to each well in a streptavidin-coated plate, and incubated at room temperature for 30 min. After washing three times with 200 µL of 20 mM potassium phosphate (pH 8.0)/0.1 M NaCl/0.05% Tween 20, 150 µL of the assay buffer was added to each well. Absorbance at 340 nm was monitored at appropriate time points by the microplate reader.

## Supporting Information

Figure S1 **Solvent accessibility of pEthF incorporation sites, V43 (A) and F179 (B), and their neighboring residues calculated by the ASA-View program.** Relative values of absolute surface area of each residue were derived from the crystal structure of the mDHFR (PDB ID: 3D80).
(TIF)

Figure S2 **Dye labeling of the mDHFR-43pEthF and the mDHFR-43biotin through CuAAC.** As a control, reactions were also performed in the absence of copper ions. Protein concentration was 30 µM. Reactions were stopped by 10 mM EDTA at 15 min after initiation, and analyzed by SDS-PAGE. The gel was illuminated by UV (365 nm) to excite the fluorophore (Fluorescence panel), and then stained with Coomassie Brilliant Blue (Coomassie panel) to visualize proteins.
(TIF)
