# Supplementary figures and images for: Site-Specific Bioconjugation of a Murine Dihydrofolate Reductase Enzyme by Copper(I)-Catalyzed Azide-Alkyne Cycloaddition with Retained Activity
Source: PLoS One. 2014 Jun 2;9(6):e98403. doi: 10.1371/journal.pone.0098403 (PMC4041766; doi:10.1371/journal.pone.0098403)

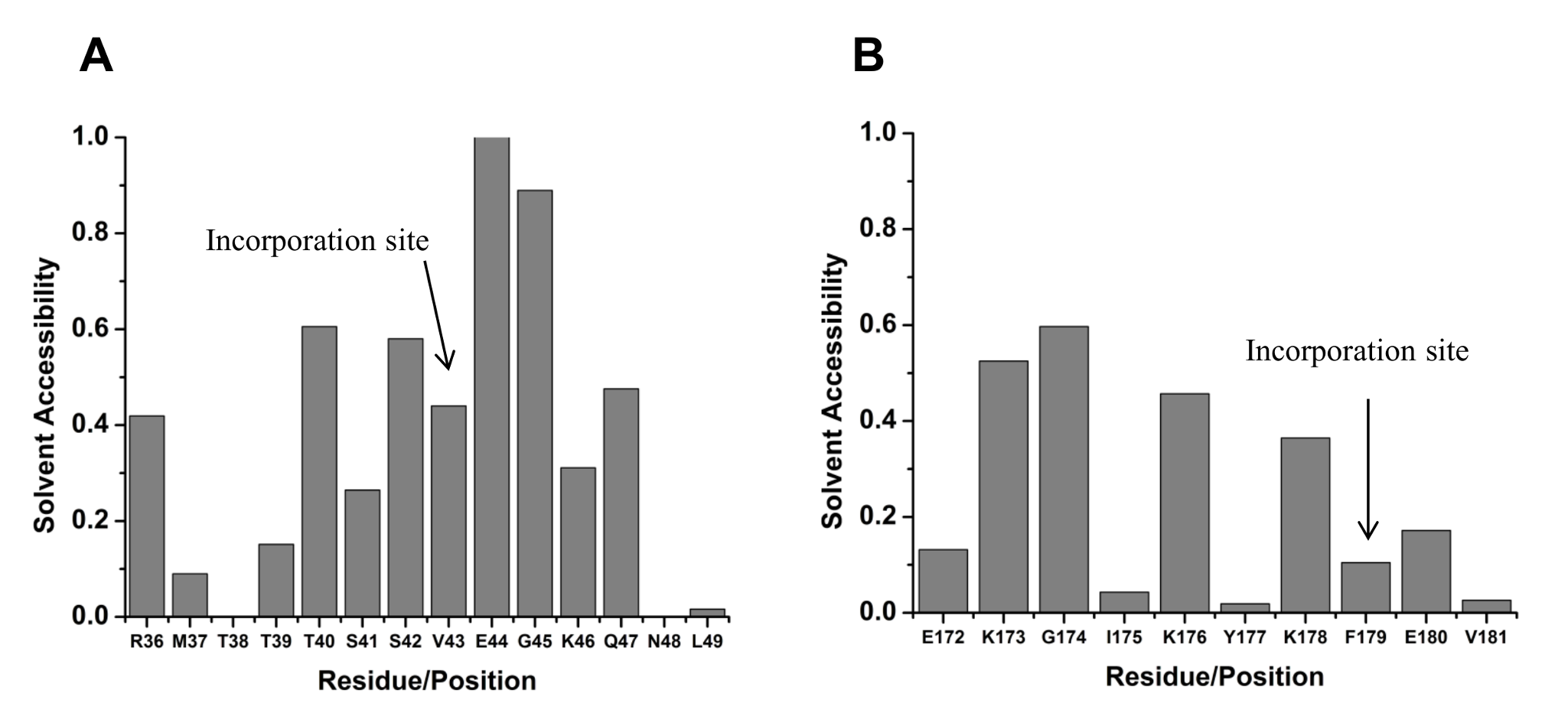

Supplement: Figure S1 — Solvent accessibility of pEthF incorporation sites, V43 (A) and F179 (B), and their neighboring residues calculated by the ASA-View program. Relative values of absolute surface area of each residue were derived from the crystal structure of the mDHFR (PDB ID: 3D80). (TIF) [file pone.0098403.s001.tif]

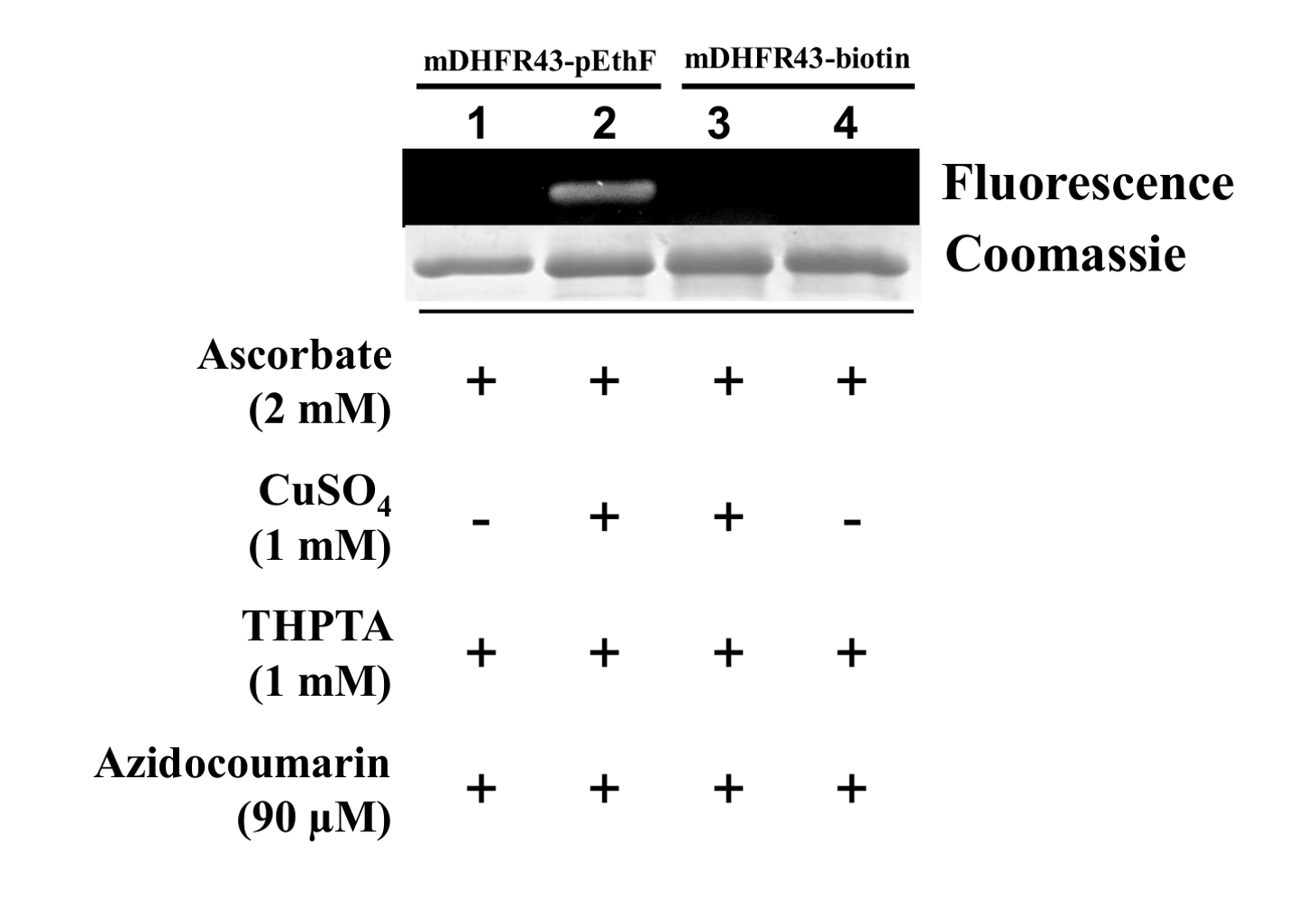

Supplement: Figure S2 — Dye labeling of the mDHFR-43pEthF and the mDHFR-43biotin through CuAAC. As a control, reactions were also performed in the absence of copper ions. Protein concentration was 30 µM. Reactions were stopped by 10 mM EDTA at 15 min after initiation, and analyzed by SDS-PAGE. The gel was illuminated by UV (365 nm) to excite the fluorophore (Fluorescence panel), and then stained with Coomassie Brilliant Blue (Coomassie panel) to visualize proteins. (TIF) [file pone.0098403.s002.tif]
